# Assessing the Diagnostic Values of the Neutrophil-to-Lymphocyte Ratio (NLR) and Systematic Immunoinflammatory Index (SII) as Biomarkers in Predicting COVID-19 Severity: A Multicentre Comparative Study

**DOI:** 10.3390/medicina60040602

**Published:** 2024-04-05

**Authors:** Anwar A. Sayed

**Affiliations:** Department of Basic Medical Sciences, Taibah University, Madinah 42353, Saudi Arabia; dsayed@taibahu.edu.sa; Tel.: +966-014-861-8888

**Keywords:** biomarkers, COVID-19, diagnostics, mortality, multicentre, NLR, Saudi Arabia, SII, systematic immunoinflammatory index

## Abstract

COVID-19 has been notoriously unpredictable in its clinical course. Such unpredictability poses a challenge to clinicians in predicting patients who will develop severe cases and possibly die from the infection. This study aims to assess and compare the diagnostic value of the NLR and SII as biomarkers in predicting COVID-19 severity, represented by mortality, with a multicentre comparative study including 855 patients in Saudi Arabia. Descriptive and analytical statistics were used to compare haematological indices between survivors and non-survivors. The median age of patients included was 41 years old, with an almost equal ratio of men to women. Most participants were Saudis, and the mortality rate in the study cohort was 13.22%. Non-survivors, as compared to survivors, were significantly older, had lower RBC counts, haemoglobin and haematocrit levels, as well as significantly higher WBC and neutrophil counts. Both the NLR and SII were capable of differentiating between survivors and non-survivors, with the latter having significantly higher values. However, the NLR was superior to the SII in such differentiation, as it had a larger area under the curve. This study further confirms the diagnostic values of the NLR and SII as biomarkers in predicting COVID-19 severity and mortality, with the NLR being more sensitive and specific. Clinical guidelines on managing COVID-19 cases should benefit from these findings by harnessing the value of the NLR in COVID-19 management.

## 1. Introduction

Four years ago, the world was faced with its first pandemic of the 21st century, with the novel coronavirus disease 2019 (COVID-19) spreading all over the world [1]. In response, countries took unprecedented measures to tackle the spread of the infection, including Saudi Arabia. Saudi Arabia took a series of progressive public measures, including halting public religious activities, travel bans and 24-h curfews [2].

COVID-19, even after 4 years of its first declaration as a pandemic and the end of its pandemic status [3], remains a medical dilemma for physicians. When it was first described, COVID-19 was described as a respiratory virus, affecting mainly the respiratory system [4]. However, subsequent studies found that the infection extends beyond the respiratory system, causing haematological abnormalities, leading to thrombotic abnormalities [5]. Furthermore, the condition was described to lead to hyperactivation of the immune system, leading to subsequent harmful autoimmune reactions damaging other parts of the body, such as the kidneys and the heart [6,7].

Another unsolved issue with COVID-19 is its unpredictable course. The pathophysiology of COVID-19 remains extremely heterogeneous, and, hence, efforts were made to develop tools to predict its course [8]. However, most of these predicting tools depend on clinical presentations and parameters that may differ significantly between countries. Thus, the applicability of these clinical tools remains limited to the geographical location in which these tools were developed.

Several studies proposed novel biomarkers that depend on haematological parameters to predict the course of COVID-19. These include the neutrophil-to-lymphocyte ratio (NLR) [9], systemic immune-inflammatory index (SII) and, more recently, the COMPred indicator [10]. Most of these studies focused on a particular tool, which makes the comparison between these tools difficult as the study cohorts were different. This study aims to address this gap in the literature and compare the NLR and SII in predicting the course of COVID-19 and, more specifically, its associated mortality. 

## 2. Materials and Methods

### 2.1. Study Design and Setting

This is a multicentre cross-sectional retrospective study that included three tertiary hospitals in Saudi Arabia. This study included 855 patients who were hospital-admitted with a suspicion of COVID-19. The diagnosis of COVID-19 was confirmed using a quantitative polymerase chain reaction (qPCR) test of a nasopharyngeal swab. Patients were admitted to the hospitals between June 2020 and December 2021. Patients were admitted based on the recommendation of the Saudi Ministry of Health (MoH) criteria for COVID-19 patients [11]. In brief, patients with any one of the following symptoms were to be admitted: clinical evidence of pneumonia, age older than 65 years, low oxygen saturation on a pulsometer of less than 94% in room air, acute respiratory distress syndrome, chronic pulmonary or kidney disease and a history of comorbidities or morbid obesity (BMI equal or more than 40). Those who yielded a negative COVID-19 diagnosis or had missing information were not included in this study.

The management of these admitted patients was consistent between the three hospitals as the national COVID-19 management protocol, i.e., the Saudi MoH for Patients Suspected of/Confirmed with COVID-19 [11], was followed throughout the course of their management. Patients who were admitted to the intensive care unit (ICU) were managed according to the Saudi MoH Protocol for Patients Suspected of/Confirmed to have severe COVID-19 [11]. These patients showed clinical signs of pneumonia, e.g., fever, cough, dyspnea and one of the following: respiratory rate more than 30/min, oxygen saturation on pulsometer less than 93% in room air or severe respiratory distress.

All of the included patients had a series of laboratory investigations performed on them, and some were repeated over the course of their hospital stay. To ensure the consistency of the compared parameters, only the first readings of their investigations were included in this study, which were conducted upon admission. Venous blood samples were collected from patients for analysis as a part of their in-hospital management. Venous blood samples were tested for a complete blood count using the Mindray BC-3200. Auto Hematology Analyzer (Shenzhen Mindray Bio-Medical Electronics Co., Ltd., Shenzhen, China). The collected parameters were based on the complete blood count investigation, which includes the red blood cell (RBC), haemoglobin level, mean corpuscular volume (MCV), mean concentration of haemoglobin (MCH), the platelet count, the white blood cell (WBC) count, neutrophil count, lymphocyte count, monocyte count and eosinophil count. The NLR was calculated by dividing the neutrophil count by the lymphocyte count, and the SII was calculated by multiplying the platelet count by the NLR.

### 2.2. Statistical Analysis

Categorical data, such as gender and nationality, was described using absolute numbers and percentages. Descriptive statistics were used to describe numerical data, such as haemoglobin levels and the NLR, in accordance with their distribution. The Shapiro–Wilk test was used to determine the data distribution in order to determine whether the data follow a normal Gaussian distribution (parametric) or not (nonparametric). Means and standard deviations were used to describe parametric data, whereas medians and interquartile ranges were used for nonparametric data. Analytical statistics were used to compare the different data sets, including the *t*-test for parametric data and the Mann–Whitney U test to compare nonparametric data.

A receiver operating characteristic (ROC) curve analysis was made, and the area under the curve was calculated to determine the indicator’s sensitivity and specificity of both the NLR and the SII. A *p*-value of less than 0.05 was considered statistically significant. A data analysis was carried out using GraphPad Prism Version 10.1.2 for Windows. 

### 2.3. Ethical Considerations

No personal or sensitive patient information was collected as part of this study. Collected data were stored on a password-protected drive and was backed up on a password-protected server. This study was conducted in accordance with the declaration of Helsinki and ethically approved by the Taibah University College of Medicine Research Ethics Committee no. TU-21-010 on 10 February 2022. Given the retrospective nature of this study, the patients’ consent was waived by the approving IRB ethics committee.

## 3. Results

### 3.1. Participant Characteristics

The analysis encompassed a total of 855 patients within the clinical cohort. The average age of patients was determined to be 41 years, with an interquartile range spanning from 27 to 57 years, indicating a relatively homogeneous distribution across age groups. Among the patients, 426 were identified as male, while 429 were female, suggesting a nearly equal gender distribution within the cohort. In terms of nationality, 550 patients were Saudi nationals, whereas 305 patients were non-Saudis, including but not limited to other nationalities such as Egyptians and Sudanese Indians. 

The examination of admission outcomes revealed notable disparities within the cohort. A total of 742 patients were discharged alive, constituting the majority of the cohort’s population. Conversely, 113 patients experienced mortality during their hospital stay, indicating a mortality rate of 13.22% within the cohort. These characteristics are described in Table 1.

### 3.2. Patients’ Baseline Investigations

The investigation into haematological parameters revealed significant insights into the blood profiles of the study population. Analysis of red blood cell (RBC) count demonstrated a median value of 4.74 (4.35–5.13) × 10^6^/mL, which falls within the reference ranges for both genders. Haemoglobin levels’ median value was at 13.40 (11.99–14.60) g/dL, similar to the RBC count, falling within the reference range for males and females. Additional parameters such as mean corpuscular volume (MCV), mean corpuscular haemoglobin (MCH), platelet count, white blood cell (WBC) count, and differential leukocyte counts were also assessed and are described in Table 2. 

Interestingly, the median readings of the laboratory investigations of the study participants were within the reference levels.

### 3.3. Non-Survivors Demonstrate a Different Haematological Profile Compared to Survivors

In examining the characteristics and investigations of survivors (n = 742) compared to non-survivors (n = 113), notable distinctions were observed. Survivors exhibited a significantly younger median age of 38 years (27–52), contrasting starkly with non-survivors, who had a median age of 62 years (49–75.50) (*p* < 0.0001). Gender distribution showed no significant difference between survivors and non-survivors, with 361 males and 381 females among survivors and 65 males and 48 females among non-survivors (*p* = 0.08). While red blood cell (RBC) counts, haemoglobin levels, haematocrit percentages, mean corpuscular volume (MCV), mean corpuscular haemoglobin (MCH) and platelet counts did not display substantial variations between the two groups, white blood cell (WBC) counts, neutrophil counts, lymphocyte counts and eosinophil counts exhibited significant differences (*p* < 0.0001). Survivors demonstrated lower median WBC counts (5.96 × 10^3^/mL) compared to non-survivors (8.33 × 10^3^/mL), along with lower neutrophil and lymphocyte counts, indicating potential immune response variations between the groups. Eosinophil counts also varied significantly (*p* = 0.002), with survivors presenting higher median counts (0.04 × 10^3^/mL) than non-survivors (0.02 × 10^3^/mL). Despite the significant differences in the haematological parameters between survivors and non-survivors, they were still within the reference range of these parameters. These findings underscore the relevance of age and immune response indicators in predicting patient outcomes, providing valuable insights for clinical prognosis and management strategies. These results are summarised in Table 3.

### 3.4. The Value of Using the Neutrophil-to-Lymphocyte Ratio (NLR) and the Systematic Immunoinflammatory Index (SII) to Discriminate between Survivors and Non-Survivors

**Figure 1:**
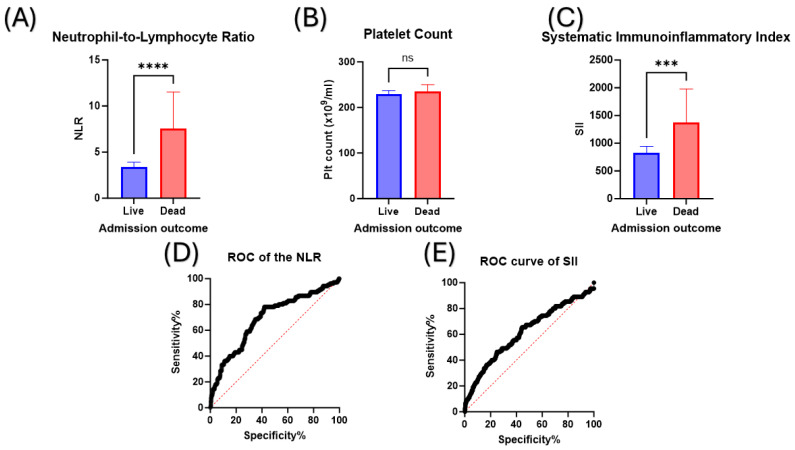
Comparison of key parameters between survivors and non-survivors. (**A**) A comparison between the NLR of patients who survived (blue column) and to those who passed away (red column). (**B**) Comparisons of platelet count and (**C**) the systematic immunoinflammatory index between survivors and non-survivors. The ROC curves of (**D**), the NLR and (**E**) the SII, demonstrating their sensitivity and specificity in differentiating between survivors and non-survivors. NLR: Neutrophil-to-Lymphocyte Ratio, ns: Non-significant, Plt: Platelet, ROC: Receiver Operating Curve, SII: Systematic Immunoinflammatory Index. *** denotes a statistically significant difference at a *p*-value < 0.001. **** denotes a statistically significant difference at a *p*-value < 0.0001.

As the median of all the haematological parameters of both survivors and non-survivors were within the reference range, it was important to develop a tool that could differentiate between survivors and non-survivors. The NLR, which depends on both the neutrophil and lymphocyte counts, can be used for such a purpose. Upon comparing the NLR between survivors and non-survivors, non-survivors had a significantly higher NLR compared to survivors (*p*-value < 0.0001; Figure 1A). The SII depends on both the platelet count and NLR to be calculated. In this study cohort, both survivors and non-survivors had comparable platelet counts (Figure 1B). However, the SII showed a statistically significant difference when compared between survivors and non-survivors, with the latter having a higher SII (*p*-value < 0.001; Figure 1C).

Next was to evaluate and compare the value of these tools, the NLR and SII, as prognostic tools for COVID-19 mortality. Using the ROC analysis, the NLR demonstrated a larger area under the curve of 0.69 (0.6334 to 0.7510 95% confidence interval; *p*-value < 0.0001) (Figure 1D) compared to the SII of 0.61 (0.5555 to 0.6809 95% confidence interval; *p*-value 0.0001) (Figure 1E). The discriminatory cutoff values for the NLR and SII were 5.3 (sensitivity and specificity > 66%) and 1051 (sensitivity and specificity > 58%), such values indicating that the NLR is superior in its sensitivity and specificity in predicting COVID-19 mortality when compared to the SII.

## 4. Discussion

Clinically, COVID-19 has always been a challenge to manage, especially given its unanticipated clinical course. Hence, the need arose among clinicians to develop predictors of disease severity upon first encounter with patients. However, most of these predictors were based on clinical examination, which may vary across physicians and populations, and some were based on a theoretical basis, such as the presence of comorbidities or in immunocompromised subjects whose immune system may not be capable of mounting a proper immune response. 

In this study, age has been identified as a risk factor for mortality among COVID-19 patients. It is not surprising, the find of the low mortality rate of over 13% among the study cohort, given that the majority of survivors were younger than 40 years of age. This rate is significantly lower than in other countries [12] and could be partially attributed to the age of this study cohort. Ageing is associated with a series of biological changes that consequently lead to a reduction in the capability of the immune system to respond properly to infections [13]. As immune competency deteriorates with age, it is no surprise that it is constantly considered a risk factor, especially among subjects older than 45 years old, based on the current literature [14].

COVID-19 is considered a proinflammatory condition, hence, haematological indices are expected to change accordingly. This gives the basis for the use of haematological parameters as a monitoring tool. This study builds on the previous extensive work that validated the use of the NLR in the context of COVID-19. Our previous multicentre study in Saudi Arabia demonstrated the prognostic value of the NLR in detecting severe COVID-19 cases, i.e., admitted to the ICU, compared to both non-severe cases and non-COVID-19 patients [9]. Such a finding was later confirmed and was found to be applicable to other cohorts locally [15,16] and internationally [17,18,19]. More recently, the works of Regolo et al. demonstrated the value of the NLR as an independent predictor of mortality in separate studies including over 1000 patients with COVID-19 [20,21]. A proper immune response requires a complex interaction between cells of the innate and adaptive immune systems. Hence, the NLR represents both of these systems, neutrophils (innate immune system) and lymphocytes (adaptive immune system). So, the changes in the NLR represented by significantly high levels of the NLR among non-survivors may reflect a sign of deranged interplay between these systems [22]. This could be in the form of hyperactivation of the innate immune system, leading to a significant rise in the neutrophil count. On the other hand, the engagement and consumption of the lymphocytes, both T and B cells, leading to a significant reduction in the lymphocytic count, could be an indicator of COVID-19 severity and, subsequently, mortality. Severe forms of COVID-19 that lead to mortality have been described to be of significantly high viral load and reduced adaptive immune response, namely, T and B cell responses [23]. In this study, the NLR had the capacity to predict mortality on its own and remains an invaluable tool in the management of COVID-19 cases given its wide availability and cost-effectiveness in comparison to other laboratory investigations [24].

Platelets are well known for their role in haemostasis. However, in more recent years platelets were found to play an important role in immunity against viral infections and have the capacity to directly interact with immune cells [25]. In the context of COVID-19, several mechanisms were suggested that could lead to a reduced platelet count as part of the COVID-19 pathophysiology. In some patients, COVID-19 lead to a cytokine storm, a state of hyperinflammatory milieu, which would subsequently reduce the production of platelets [7,26]. Another alternative mechanism of reduced platelet count could be attributed to their increased destruction by autoantibodies as a side effect of SARS-CoV-2 infection [27]. Despite the safety and effectiveness of COVID-19 vaccines [28], several reports have suggested that COVID-19 vaccines could have the adverse reaction of a reduced platelet count [29]. Such an adverse reaction has been attributed to an autoimmune response that is triggered by adeno-vector vaccines such as the AstraZeneca vaccine, leading to a case referred to as vaccine-induced immune thrombotic thrombocytopaenia (VITT) [30]. VITT was explained to be due to an interaction between antiplatelet factor 4 (PF4) antibodies and platelets, leading to their destruction through Fc receptors and opsonisation by phagocytes. Another possible mechanism is that these PF4 antibodies activate platelets causing them to start the thrombotic cascade, and hence their consumption in the process [31].

Given the value of platelets in the immune response against viruses, it was only logical to include it as a predictor of severity and incorporate it with the NLR to develop the SII [32]. In this study, platelet count was not sufficient to differentiate between survivors and non-survivors, in line with the work of Yuan and colleagues [33]. However, the SII was capable of predicting mortality, but with lesser sensitivity and specificity as compared to the NLR. Such findings confirm the previous work of Ghobadi and his team [34], Gutiérrez-Pérez and colleagues [35] and Yılmaz and her team [36], as well as in Turkey [37]. In contrast, previous studies either refuted and excluded the value of the SII in the context of COVID-19 [38] or found it to be exclusively useful and to surpass the limited value of the NLR [32]. Such contradictory findings may be explained by the limited study sample sizes, which were not sufficient to determine the value of the NLR, or the skewed study cohort by including elderly patients only. 

The primary objective of this study was to compare the diagnostic value of the NLR and SII in predicting mortality among patients with COVID-19. However, more results came out of this study that provide further insights into the pathophysiology of severe COVID-19. Patients who did not survive due to COVID-19 were significantly older than those who survived, confirming the previous findings that age is an independent risk factor for the severity of COVID-19 cases. This is due to the biological changes associated with the ageing process that lead to the gradual deterioration of the immune system, also known as immunosenescence [13]. These changes include but are not limited to a low lymphocytic repertoire (leading to an immunological response against a limited number of pathogens), shorter telomere length as well as structural changes within immune organs. Given the inverse correlation between age and the fitness of the immune response, many studies have considered it an important predictor of the severity of COVID-19 cases and include it in their COVID-19 mortality prediction tools [10,14].

Another possible side effect of COVID-19 infections is the development of anaemia. Although several mechanisms are proposed for COVID-19-related anaemia, such as via the haemolytic pathway caused as part of the COVID-19 pathophysiology [39,40], it remains a sign of severe COVID-19 infection. The findings of this study further confirm this principle, but not necessarily to the degree of overt anaemia. In this study, non-survivors had significantly lower haemoglobin levels compared to survivors. Nevertheless, the haemoglobin levels in both cohorts, despite the statistically significant difference, were within the normal reference values. Indeed, the results of Bergamaschi and colleagues on 206 COVID-19 patients found that the presence of anaemia is not a direct predictor of severity in COVID-19, but rather an indication of old age and frailty [41]. The study by Mi Oh and colleagues showed that anaemia upon admission increased the odds of all-cause mortality among COVID-19 patients, with anaemia of haemoglobin of less than 11 g/dL considered an independent risk factor [42]. While such a conclusion may oppose the findings of this study, it is important to consider the age factor, as the median age in this study cohort was 41 years old, whereas in Mi Oh’s study, it was 65 years old. Hence, anaemia, as defined by haemoglobin below the reference range, could be a predictor of COVID-19-related severity, and possibly mortality, in elderly patients.

Despite the merits of this study of providing new insights to the current scientific literature, this study is not without limitations. Firstly, this study is a retrospective cross-sectional study, which is at risk of sampling bias [43]. Secondly, this study did not consider other influencing factors, such as the presence of comorbidities and smoking status, which may significantly affect the mortality associated with COVID-19. Lastly, this study focused merely on values obtained and derived from an ordinary CBC. However, this does not negate the importance of other haematological markers that have shown their value in predicting COVID-19-related mortality, such as C-reactive protein (CRP) and interleukin (IL)-6 [44,45].

Future studies should aim to further build on the findings of this study and address the limitations of this study. For example, a prospective multicentre study with more comprehensive data should be conducted to assess the sensitivity and specificity of these indicators, the NLR and SII.

## 5. Conclusions

In conclusion, this study presents both tools, the NLR and SII, as valuable assets that can be used by clinicians to predict COVID-19 severity. These tools are affordable, widely available and rapid, which should facilitate the decision-making process when managing the uncertainties associated with COVID-19 cases. These tools, the NLR more specifically, should be incorporated into medical guidelines on the management of COVID-19. 

## Figures and Tables

**Table 1:** Patients’ Characteristics.

Characteristic (Unit)	Values
Age (years)	41 (27–57) *
Gender	Male: 426
	Female: 429
Nationality	Saudi: 550
	Non-Saudi: 305
Admission outcome	Alive: 742
	Deceased: 113

* Years are expressed as median (interquartile range).

**Table 2:** Patients’ laboratory investigations at baseline.

Investigation (Unit)	Patients’ Readings	Reference Levels
RBC count (×10^6^/mL)	4.74 (4.35–5.13)	Male: 4.0–5.9
		Female: 3.8–5.2
Haemoglobin (g/dL)	13.40 (11.99–14.60)	Male: 13.8–17.2
		Female: 12.1–15.1
Haematocrit (%)	40.70 (37.13–44.10)	Male: 40–54
		Female: 36–48
MCV (fl)	86.25 (81.53–89.80)	80–100
MCH (pg)	28.60 (26.80–30)	27–31
Platelet count (×10^6^/mL)	229.5 (182–290.5)	150–450
WBC (×10^3^/mL)	6.24 (4.57–9.29)	4–11
Neutrophil count (×10^3^/mL)	4.28 (2.71–7.49)	2.5–7
Lymphocyte count (×10^3^/mL)	1.07 (0.69–1.59)	1–4.8
Monocyte Count (×10^3^/mL)	0.33 (0.23–0.48)	0.2–0.8
Eosinophil count (×10^3^/mL)	0.04 (0.01–0.08)	0.03–0.35

All values are expressed using the median value (interquartile range).

**Table 3:** Comparison of patients’ characteristics and lab investigations between survivors and non-survivors.

Characteristics/Investigation (Unit)	Survivors (n = 742)	Non-Survivors (n = 113)	*p*-Value
**Age (years)**	**38 (27–52)**	**62 (49–75.50)**	**<0.0001**
Gender	Male: 361	Male: 65	0.08 ^
	Female: 381	Female: 48	
RBC count (×10^6^/mL)	4.76 (4.37–5.14)	4.60 (4.09–5.08)	0.09
**Haemoglobin (g/dL)**	**13.50 (12.20–14.70)**	**12.88 (11.60–14.35)**	**0.007**
**Haematocrit (%)**	**41 (37.30–44.20)**	**39.70 (35.70–43.30)**	**0.046**
MCV (fl)	86.20 (81.70–89.80)	86.70 (80.90–89.90)	0.73
MCH (pg)	28.70 (26.90–30)	28.10 (26.40–29.90)	0.23
Platelet count (×10^6^/mL)	228.5 (185–294)	235 (160.9–285)	0.46
**WBC count (×10^3^/mL)**	**5.96 (4.45–8.56)**	**8.33 (5.78–11.60)**	**<0.0001**
**Neutrophil count (×10^3^/mL)**	**3.93 (2.57–6.63)**	**6.16 (4.16–9.86)**	**<0.0001**
**Lymphocyte count (×10^3^/mL)**	**1.13 (0.74–1.66)**	**0.76 (0.53–1.25)**	**<0.0001**
Monocyte Count (×10^3^/mL)	0.33 (0.23–0.48)	0.33 (0.20–0.53)	0.32
**Eosinophil count (×10^3^/mL)**	**0.04 (0.01–0.08)**	**0.02 (0.00–0.06)**	**0.002**

All values, except for gender, are described as a median (interquartile range). All comparisons, except for gender, were performed using the Mann–Whitney U test, given the nonparametric distribution of the data. Values written in bold denote statistically significant differences between survivors and non-survivors. ^ Chi-square test was used.

## Data Availability

The data presented in this study are available on reasonable request from the corresponding author.
